# Supplementary material for: Are carbon emissions trading and green financial instruments synergistic? -Comprehensive quantitative research based on content analysis
Source: PLoS One. 2024 Mar 7;19(3):e0298601. doi: 10.1371/journal.pone.0298601 (PMC10919601; doi:10.1371/journal.pone.0298601)
Supplement: S1 Data — (ZIP) [file pone.0298601.s001.zip › Data/地级市Z_F工作报告.zip!/╔┬╬≈/╧╠╤⌠╩╨/└┤╫╘╣1⁄2╓┌║┼í╛┬φ┐╦╩2╛▌═°í┐_2015.docx]

各位代表：

　　现在，我代表市人民政府，向大会作工作报告，请予审议，并请市政协委员和其他列席人员提出意见。

　　一、2014年工作回顾

　　过去一年，我们深入贯彻党的十八届三中、四中全会精神，牢牢把握“稳中求进、改革创新”的总基调，积极应对宏观经济形势趋紧、下行压力加大和结构调整阵痛，统筹推进稳增长、调结构、抓改革、促开放、优环境、惠民生各项工作，主要经济指标增速位居全省第一方阵前列，生产总值增长10.9%，高出全省1.2个百分点，成功跨过2000亿元大关；财政总收入和地方财政收入分别完成210.2亿元、85.3亿元，增长10.3%、16.2%；预计城镇居民收入和农民人均纯收入分别达到31480元、9605元，增长10.5%、12.5%，城镇登记失业率控制在3.2%以内。

　　一年来，我们主要抓了九个方面的工作。

　　（一）坚持扭住关键、精准发力，工业经济稳步增长。先后两次出台稳产促销保市场政策措施，加强生产要素保障，大力实施工业和非公经济两个“1113”工程，工业经济实现稳步回升。强化银政企对接，建立担保贷款风险补偿金制度，全市新增贷款余额139.5亿元，有效缓解了企业融资难题。彬长旬清洁能源基地、高新区、纺织工业园等重点园区加快建设。实现规模以上工业增加值912.8亿元、增长14.5%。工业的持续发展，增加了税收，扩大了就业，为加快城镇化提供了有力支撑。

　　（二）坚持夯实基础、健全体系，现代农业加快发展。全年粮食总产184万吨、水果562万吨、蔬菜412万吨、肉蛋奶115万吨。新增省级现代农业园区12家、市级以上龙头企业10家。总投资36亿元、年屠宰生猪168万头的冷鲜肉深加工项目进展顺利。泾河东庄水库获国家正式批复，亭口水库、红岩河水库等重点项目完成节点任务。国家农业机械化示范区建设扎实推进，气象防灾减灾能力有效提升，农业基础地位更加稳固。

　　（三）坚持打造特色、提档升级，现代服务业加速崛起。电子商务加快发展，苏宁云商、西域美农等一批电商企业成功入驻，当当网项目签约，武功进入省级电子商务示范县，全市电子商务营业额达到60亿元。启动国家创新型城市建设，技术合同交易额和专利授权量分别增长10.3%、23.4%，被确定为国家知识产权试点城市。以袁家村、马嵬驿为代表的乡村旅游极具人气，彬县大佛寺石窟入选世界文化遗产名录。社会消费品零售总额完成525.2亿元、增长14.3%，增速位居全省第一。

　　（四）坚持战略招商、项目带动，投资保持快速增长。投资50亿元的煤制乙二醇、25亿元的正泰电气产业园等重点项目加快推进，投资130亿元的华晨汽车产业园开工建设。全市150个重点项目完成投资866.7亿元，占年计划的126.4%。西咸新区50个重点项目完成投资164.2亿元、占年计划的140.6%。全年共引进合同项目251个，10亿元以上58个，完成投资750亿元。实现全社会固定资产投资2492.4亿元、增长21.3%。项目在扩增量、优结构、促转型中发挥了重要作用。

　　（五）坚持完善功能、提升品质，统筹发展步伐加快。全面融入大西安，北客站到空港的轻轨项目开工建设，地铁一号线二期启动勘察设计，西咸北环线加快推进，大西安文化体育功能区建设开局良好。咸旬高速建成通车，实现了县县通高速的目标。年度投资139.4亿元的192个城建项目扎实推进，新修和改造道路27.7公里，城西快速干道建成通车，拉大了城市框架。人民广场、文咸广场改造完工，咸阳植物园、丝路公园等13项园林绿化工程进展顺利。扎实开展多城同创和城乡环境综合整治，荣获国家园林城市称号，实现省级园林县城全覆盖。跻身国家信息惠民试点城市和全国十大智慧城市。新型城镇化规划编制完成，16个省市级示范镇、文化旅游名镇完成投资24.8亿元。21个新型农村社区、24个传统民俗文化村和16个农村改革试验点建设取得阶段性成果。

　　（六）坚持铁腕整治、系统推进，生态环境持续改善。扎实推进“治污降霾·保卫蓝天碧水”行动，总投资12.1亿元的13大类污染治理项目完成555个。提前一年完成渭河流域水污染防治三年行动任务，彻底结束了污水直排渭河的历史。下决心关闭了严重影响城市环境的太西氮肥厂，拆改燃煤锅炉303台，淘汰黄标车和老旧机动车1.2万辆，全面启动湿地建设，完成营造林38万亩。全年空气质量二级和好于二级天数超出省考指标76天。

　　（七）坚持民生为先、办好实事，社会保持和谐稳定。投资100亿元的35件惠民便民实事全面完成。提高城镇基本医疗保险和新农合保障水平，将儿童白血病、儿童先天性心脏病等22种大病报销全部纳入新农合保障范畴。新开工保障性住房3.8万套，实现稳定脱贫11.8万人，为11.5万户、28.3万人发放最低生活保障金5.7亿元。把卫生、教育、文化和体育事业放在更加突出位置，制定相关规划，促进布局优化、服务均等。“双高双普”工作扎实开展，武功、三原通过省政府评估验收，大职教格局初步形成。成功申办省第十六届运动会，市民文化中心的青少年宫、科技馆等八大场馆全面封顶，大秦剧院投入使用，中小学校体育运动设施向市民开放。新增100辆公交车和1000辆公共自行车。食品药品监管工作有效加强，“放心早餐”经营网点逐步扩大。引石过渭倒虹段除险加固工程完工，市区供水安全得到保障。平安建设深入推进，安全生产“三化”建设和农村道路交通管理“咸阳模式”在全省推广。

　　（八）坚持勇于创新、重点突破，各项改革有力推进。着眼优政惠民，完成了48个市级部门的行政审批事项精简任务，取消76项，下放35项，精简率36.6%。集体土地所有权登记基本完成，农村承包地经营权确权登记颁证、医药卫生体制、城乡义务教育等改革取得实质性进展，农业农村、职业教育、殡葬制度等多项改革走在全省前列。工商登记制度改革在全省率先实施，全市新增市场主体2.7万户，增长22.9%。通过这些措施，激活了市场、方便了群众，支持改革、推动改革已经成为全市人民的共同认识和自觉行动。

　　（九）坚持践行宗旨、改进作风，政府效能全面提升。市政府班子群众路线教育实践活动各项整改措施全面落实，制定了调查研究、厉行节约等5大类17项制度。坚持问题导向，针对群众反映强烈的克扣惠民资金、执法监管部门“三乱”等问题进行集中整治，查处违规案件13起、党政纪处分13人。坚决执行“八项规定”，“三公”经费压缩13.6%，拍卖超标超配公务车270辆，清理清退办公用房3.5万平方米，干部作风明显转变，“五型”政府建设取得新成效。

　　双拥、老龄、慈善、残疾人、红十字等事业全面发展，外事、侨务、对台、审计、统计、民族、宗教、地震、人防、地方志等工作有了新进步。

　　各位代表，过去一年，宏观经济形势严峻复杂，我市发展稳中有进、逆势前行，成绩实属来之不易。这是省委、省政府和市委坚强领导的结果，是全市广大干部群众团结奋斗的结果。在此，我代表市政府向全市人民，向各位人大代表、政协委员，向驻咸部队、武警消防官兵和政法干警，向所有关心支持咸阳发展的各界人士表示衷心的感谢！

　　在肯定成绩的同时，我们也清醒地认识到，咸阳地处欠发达地区，发展不足的市情短期内还难以改变，经济总量不大与质量不高并存，一些长期存在的老问题和转型发展的新矛盾相互交织。主要表现在：产业结构不合理，城乡发展不协调，城镇化率低于全国、全省平均水平；环境保护压力大，节能减排形势严峻；人均财力弱，社会事业投入欠账较多；政府职能转变有待深化，依法行政能力亟需提高。这些问题，我们将高度重视，在今后工作中认真加以解决。

　　二、2015年工作总体要求和预期目标

　　2015年，是“十二五”规划的收官之年、全面推进依法治市的开局之年和深化改革的关键之年，做好今年政府各项工作，任务艰巨、责任重大。当前，我国经济发展步入速度变化、结构优化、动力转化的新常态，这一趋势性变化对我们做好经济工作提出了新的要求。展望未来，2018年我们将举办省运会，2020年我们要与全国全省同步够格进入小康社会，对咸阳经济社会发展提出了更高的标准。这些不仅要贯穿于今年经济工作始终，而且要作为编制“十三五”规划的基本遵循。

　　深刻认识和准确把握新常态，既要正确看待当前经济下行、结构调整带来的压力和挑战，更要看到国家大战略布局调整带来的有利机遇和动力，全力推进提质增效、加快发展。随着西部大开发和丝绸之路经济带建设深入实施，作为关中城市群和大西安的核心区，作为国家确定的资源成长型城市和创新型试点城市，加之西咸新区成为国家级新区，咸阳在全省乃至西部发展的战略地位更加突出，政策和区位优势叠加放大，历史性地将咸阳推到了创新驱动、开放开发的中心地带和重要位置。特别是多年来经过全市上下的共同努力，我市农业产值稳居全省第一，工业七大支柱产业支撑经济高速平稳增长，以及日益壮大的县域经济规模，都为我们实现雁行破阵、率先领跑奠定了基础。我们必须紧紧抓住这些机遇和有利条件，大力实施改革促发展、开放促发展、转型促发展、共建促发展“四促举措”，更加注重加强项目建设带动经济增长，充分发挥投资、消费和出口的支撑作用，推动转型升级不断取得新进展；更加注重深化改革开放释放发展潜力，通过创新驱动培育新的增长点；更加注重增进民生福祉，以创建全国文明城市促进生态环境优化、城市品质提升和人民生活改善，不断开创经济社会发展的新局面。

　　2015年政府工作的总体要求是：全面贯彻落实党的十八大和十八届三中、四中全会、省委十二届六次全会和市委六届十次全会精神，坚持“稳中求进、改革创新”的工作总基调，立足新常态、运用新思维、谋求新突破、实现新跨越，统筹抓好保增长、转方式、调结构、促改革、补短板、惠民生、防风险各项工作，全力推动经济实现中高速增长、发展迈向中高端水平。

　　主要预期目标是：生产总值增长11%左右；全社会固定资产投资增长22%；规模以上工业增加值增长14.5%左右；地方公共财政预算收入增长12%；社会消费品零售总额增长14%左右；城镇居民人均可支配收入增长11%；农民人均纯收入增长12%。

　　各位代表，确定适度加压的发展目标，是立足于咸阳多年来增速位居全省第一方阵前列，为全省发展贡献正能量的需要；是兑现全面建成小康社会庄严承诺，为转型发展争取时间、赢得空间的需要。只要全市上下盯紧目标，共同努力，困难面前不退缩，激流勇进争上游，就一定能够迈出建设"四强三领先"西部强市的新步伐！

　　三、2015年主要工作任务

　　（一）以工业为支撑打造咸阳经济升级版

　　坚持工业主导不动摇，以2020年工业总量翻番为目标，以产业集群集聚发展为方向，以两个"1113"工程为抓手，全力推进七大支柱产业加速发展。

　　充分释放现有产能。强化服务意识，严格实行"领导联系、部门负责、一企一策"工作机制，力争大企业大集团实现产值1100亿元以上。强化要素保障，认真落实工业稳增长16条、新13条措施和煤炭企业减免收费政策，用足用好供电供气直接交易等便利政策。强化引导支持，促进中小企业做大做强，年内新增规模以上工业企业60户，工业产销率达到96%以上。

　　培育壮大产业集群。做好顶层设计，明确路径措施，突出重点产业、重点板块，深度谋划实施七大支柱产业转型升级计划，走集群化发展路子。突出煤化工和精细化工，支持橡胶产业联盟创新发展，加快能化产业向高端化迈进。突出汽车及零部件、航空船舶、能源设备，不断壮大先进装备制造产业。突出粮油、果汁饮料、乳制品、肉制品加工，推动食品产业品牌化、链条化发展。突出大数据、新型平板显示器、电子信息材料，力推CEC8.5代液晶面板生产线上马，再造电子信息产业新优势。强化政策支持，推动新能源、新材料、新技术等战略性新兴产业加快发展。

　　全力抓好平台建设。加快"两区一基地三园区"建设步伐，强化要素整合，以龙头带产业、以产业兴园区，实现专业化、特色化发展。坚持筑巢引凤，探索建立市场化运作、多元化投入的建设机制，完善水、电、路、气等基础设施，提升园区承载能力。加大彬长旬清洁能源基地建设力度，健全工作推进机制，力争在引进大型煤化工项目上取得新突破。抓好高新区开发建设，加快医药产业聚集入园，带动医药产业做大做强。纺织工业园突出退城入园与引进企业并举、扩能升级与延伸链条并重，加快和立东升等项目建设步伐，二、三分厂尽早投产，推动园区投入产出步入良性循环。加快高科技历史文化产业园工作进度，确保上半年开工建设。建立项目联手引进和利益共享机制，优化项目布局，提升31个县域工业集中区产业结构和发展水平，加快标准化厂房和中小企业孵化器建设，着力打造县域经济发展增长极。

　　（二）以转变发展方式为主线壮大现代农业

　　加快推进农业现代化，大力发展特色农业、品牌农业，实现传统农业向优质高效、生态观光农业转变。

　　加快农业产业结构调整。围绕稳粮、优果、扩畜、增菜，在提高产能和提升品质上下功夫，积极打造肉蛋奶茶生产重点县，确保粮食总产保持在180万吨左右。大力发展双矮苹果及特色果品，新栽各类果树15万亩。创建省部级标准化示范场10个，生猪存栏达到221万头。

　　大力推进农业产业化经营。按照"板块化布局、规模化经营、标准化生产、社会化服务"的要求，努力提升农业产业化经营水平，促进一二三产融合互动。强化龙头带动和园区引领，谋划引进一批农产品深加工项目，全年新增农业产业化龙头企业15家。强化新型经营主体培育，支持种养大户、家庭农场和农民专业合作社发展，加快农村土地经营权确权登记颁证工作，引导和规范土地经营权有序流转，积极发展适度规模经营。强化农业市场体系建设，加大市场营销力度，打造特色品牌，加快"马栏红"苹果产区规模化、标准化建设，推进农业电商发展，提升咸阳农产品竞争力和市场占有率。强化农民技术培训，大力实施职业农民培育工程，完成职业技能培训25万人次，培育一批生产经营型、专业技能型、社会服务型职业农民。

　　提升农业基础保障能力。加快亭口水库、红岩河水库、柏岭寺水库、张家山泉群开发等重点水利工程建设，做好东庄水库、引汉济渭工程服务工作。加大科技投入，抓好西北农林科技大学苹果、旱作农业等5个试验站建设，推广精确施肥、节水灌溉等先进适用技术，发挥科技的引领带动作用。提升农业机械化水平，加强气象服务和防灾减灾工作。扎实有序做好农村改革和村委会换届工作，为农业农村发展提供组织保障。

　　（三）以服务业为龙头培育新的增长点

　　实施《振兴服务业三年行动计划》，在拓展领域、优化结构、提升水平上下功夫，全力打造中华养生文化名城、休闲度假城和电子商务示范城。

　　优先发展现代物流业和电子商务产业。围绕构建制造业、大宗商品、农产品、保税物流四大体系，推进咸阳航空物流基地等重大前期项目，加快苏宁云商、当当网等项目建设，打造大西安地区物流基地。制定发展规划，加快电子商务产业园区和服务中心建设，鼓励各县市区发展电子商务产业，促进电子商务与实体经济有机对接。

　　做大做强旅游和文化产业。抓好乾陵唐文化景区、汉唐帝陵旅游等项目建设，提升拓展袁家村、马嵬驿乡村旅游发展模式，加强美丽乡村建设，把400万亩果园变成大花园，吸引大西安游客休闲游、度假游、避暑游，让咸阳旅游有看头、有玩头、有买头，走了以后想回头。力争全市旅游接待人数突破4600万人次，综合收入突破250亿元。积极谋划和实施文化产业项目，促进文化与科技、信息、金融及城市建设融合发展，推动文化产业快速增长。

　　加快培育新的消费业态。依托中医药和地热资源，鼓励发展医疗、保健、养老等健康产业，培育一批中医养生和保健旅游品牌，打造西部领先、国内一流、国际知名的"健康咸阳"。发展家政服务、社区服务，满足群众多样化、个性化消费需求。优化城乡商业网点结构和布局，加快连锁超市、特色街区建设，带动消费升级。注重培育壮大研发设计、商务咨询、服务外包等新兴产业，促进高端高回报生产性服务业加快发展。

　　（四）以扩大开放为抓手提升经济外向度

　　坚持以开放促发展，主动融入国家对外开放和区域协调发展新格局，加快建设内陆开放开发新高地。

　　积极参与丝绸之路经济带和大西安建设。坚持超前谋划、统筹实施，加快西安北客站至咸阳机场轻轨、地铁一号线二期工程建设，推动高铁、高速公路等交通设施无缝对接，发挥机场综合交通枢纽作用，促进交通一体、产业共建。加快大西安文化体育功能区建设，全力推进市民文化中心和奥体中心建设，打造现代服务业、临空物流聚集区和统筹城乡发展示范区。积极服务西咸新区发展，支持空港新城国家航空城试验区和海关特殊监管区建设，支持秦汉新城第四军医大学医教研综合园区建设，支持沣西新城中国西部科技创新港和信息产业园建设，支持泾河新城国家现代农业示范区建设。加强与中亚国家的文化旅游和农业技术合作交流，鼓励企业拓展建材、食品、纺织品市场，不断扩大外贸进出口份额。

　　抓好招商引资和项目建设。把引进域外投资、激活民间资本作为扩大开放的着力点，以壮大产业规模、增强自身实力为基础，不断提升与国内外市场合作发展的水平。狠抓战略招商，严格执行"五个不要"负面清单，围绕完善产业链和培育产业集群，突出工业和高科技项目招大引强，扎实开展"一对一、点对点"精准定向招商，确保完成省上下达的810亿元到位资金任务（含西咸新区）。狠抓项目建设，确保总投资4628亿元的190个市级重点项目顺利推进，完成年度投资766亿元。狠抓环境保障，继续深化投资发展环境专项整治，严肃查处典型案件，坚决斩断伸向企业的黑手，打造营商环境，提高宜商指数，让投资商和创业者在咸阳安心发展壮大。

　　（五）以"人"的城镇化为重点推动城乡统筹发展

　　坚持把新型城镇化作为经济转型升级的强大引擎，突出"新"字、写好"人"字，以城带乡、产城融合，加快城乡一体化步伐。

　　全面提升中心城市建设管理水平。强化规划引领作用，尽快完成主城区总体规划修编工作，围绕迎接2018年省运会制定《主城区三年提升计划》，优化城市发展布局。突出完善功能与提升品质并举、丰富内涵与塑造特色并重，深入实施"3+6"工程，抓好年度重点城建项目，推进产业、交通、环境有机衔接。坚持以水为脉，加快咸阳湖二期和双照调节蓄水库立项建设，严格落实生态湿地建设规划，加大泾河、渭河湿地恢复力度，加快马栏河、清河国家级湿地公园建设，打造城市生态景观带。坚持以绿为韵，围绕创建国家绿化模范城市，大力推进"建绿、扩绿、补绿、透绿"，强化特色街区建设和街景整治，提升绿化亮化美化水平。坚持以文为魂，加强对历史街区的有机保护，保留历史文化符号，彰显城市人文底蕴。严格落实城市公共空间管理条例，积极推行数字化城管。抓好国家卫生城市复审工作，深入推进全国文明城市创建，全面提高市民文明素质。

　　加快推进县域城镇化和城乡一体化。抢抓关中城市群建设机遇，年底前完成县城总体规划调整修编，加大县城给排水、供热、供气等市政设施建设，完善教育、医疗、文化等公共服务功能。加快武功、淳化等5个县城过境公路升级改造，配合做好铜旬高速建设，改造县乡公路200公里。加快省市重点示范镇、文化旅游名镇和沿渭示范镇建设。积极创新人口管理和服务，认真做好农民工养老、医疗等社会保障和子女义务教育工作，年内农业转移人口市民化12.5万人，城镇化率达到49.5%。把移民搬迁和小村并大村有机结合起来，加强农村基础设施建设，延伸基本公共服务体系。坚持因地制宜、突出特色，加大对有历史记忆、关中特色的传统村落的保护力度，完善并继续加快新型农村社区试点和传统民俗文化村建设，让城乡居民共建共享现代文明。

　　（六）以系统治理为关键加快建设美丽咸阳

　　顺应群众对清新空气、清澈水质、清洁环境的期待，突出山水林田湖系统治理，让良好的生态环境成为最普惠的民生福祉。

　　强力推进"治污降霾·保卫蓝天碧水"行动。按照"生态、景观、安澜"的要求，启动实施新一轮渭河三年行动计划。继续推进污水处理厂建设，统筹做好管理工作，实现正常运行、达标排放。扎实推进气化咸阳，今年市区供热普及率达到80%，逐步扩大县城供热范围。加强重点行业治污设施提标改造，完成65蒸吨以上燃煤锅炉脱硝工程，年内全部淘汰2005年以前注册营运的黄标车，确保城市建成区主要车行道机扫率达到90%以上，坚决打好大气污染防治攻坚战。

　　狠抓资源节约和淘汰落后产能。坚守耕地红线和水资源开发利用控制、用水效率控制、水功能区限制纳污"四条红线"，集约节约用地，扎实做好节水、节能工作。坚决淘汰落后产能，大力发展循环经济和环保产业，健全水、气环境质量补偿考核机制。

　　加强生态修复。扎实开展"旱腰带"地区资源整合和开山采石整治工作，确保"一年打基础、两年初见绿、三年变面貌"。继续加强农村环境连片整治，实施土壤治理修复示范工程，积极防治重金属、危险化学品和危险废物污染。抓好林业重点工程建设，完成营造林30万亩，绿化道路300公里。

　　（七）以利民惠民为根本促进社会和谐

　　坚持守住底线、突出重点、完善制度、引导舆论的思路，着力保障基本民生，更加关注弱势群体，促进社会和谐稳定。

　　以创业带动就业。落实鼓励大众创业、发展壮大市场主体的《若干意见》，进一步完善就业创业政策体系。在政府机关、国有企业事业单位全面推行大学生见习制度，积极增加公益性岗位，向困难群众提供免费职业教育，深入实施农民收入倍增计划，强化劳动技能培训，确保全年城镇新增就业5万人。

　　加强社会保障。严格落实城乡居民基本养老保险《实施办法》，继续做好个体工商户、灵活就业人员参保工作，推进机关事业单位养老保险制度改革。完善社会救助体系，重视做好城市困难群众生活保障，改进城乡低保和农村五保工作，落实农村留守儿童、妇女、老年人关爱政策，健全残疾人权益保障制度，让公共财政的阳光更多温暖困难群众。

　　大力发展社会事业。严格落实全市卫生、教育、文化、体育等规划，突出抓好"双高双普"、职业教育、特殊教育和县城以上城区中小学建设，加快实施第二期学前教育三年行动计划，促进义务教育均衡发展。全面深化医药卫生体制改革，建立健全分级诊疗制度，鼓励妇幼保健和计生技术资源深度融合，为群众提供安全、有效、方便、价廉的医疗卫生服务。大力实施文化惠民工程，积极创建全民健身示范市，不断满足群众的精神文化需求。

　　加快平安咸阳建设。加强对治安混乱地区和治安突出问题排查整治，建立健全网格化管理服务体系，提高群众对社会治安的满意度。抓好"六五普法"，增强全社会法制意识。完善法律援助制度，健全人民调解与行政调解、司法调解联动机制，将信访工作纳入法制化轨道，维护群众合法权益。深入开展双拥共建。提升安全生产"三化"建设水平，确保群众生命财产安全。

　　各位代表，我们深知，保障和改善民生没有终点，只有连续不断的新起点。今年我们将继续办好30件惠民便民实事，下大气力解决群众身边的烦心事、难心事。围绕让群众吃的放心，建立农产品质量安全追溯平台，强化食品药品市场监管和城乡饮水安全保障，解决农村20万人的饮水安全问题，实现石头河供水市区全覆盖，确保人民群众"舌尖"上的安全。围绕让群众居住舒心，对旱腰带地区1370户5500人实施扶贫移民搬迁，投资60亿元建设保障性住房3.5万套，提高入住率、注重宜居性，确保公开公平公正分配。加快棚户区、老旧社区改造提升步伐，合理布局菜市场和便民超市，提升物业管理服务水平。围绕让群众出行顺心，对城市道路、背街小巷进行改造提升，启动城区公共停车场、住宅小区停车场建设，全面推行公交刷卡。围绕让群众生活安心，实施教育惠民工程，继续推进贫困寄宿生补助和农村义务教育学生营养改善计划；加快市县医院、乡镇卫生院和村卫生室建设，建设农村互助幸福院340个。围绕让群众过得开心，实施信息惠民优政工程和"光网城市"建设，突出深化应用，加快推进群众办事"一站通"、公共服务"一卡通"，实现咸阳湖景区免费WIFI全覆盖。

　　（八）以深化改革为引擎释放发展活力

　　立足新常态，必须通过深化改革释放市场潜力、激发社会活力，使改革新红利转化为发展的动力源。

　　坚持以政府自我革新带动重要领域改革。以转变政府职能、优化组织结构、创新行政管理方式为重点，加快推进简政放权。认真做好新一轮政府机构改革、镇村综合改革、事业单位分类改革，加快网上政务服务中心建设，大力推行网上审批、并联审批和集中审批，压缩审批环节三分之一以上，实现一门式办理、一站式提交。加强对行政审批的电子监察和全程开放式监管，以公开促公平、以公平促公正，努力提高行政效能和服务水平。

　　牢牢抓住市场在资源配置中起决定作用这个核心。深化工商登记制度改革，积极推行营业执照、税务登记证等"四合一"审批办理，促进市场主体快速发展。深化国有企业改革，强化市级经营性国有资产管理，实现经营管理与资本管理分离，确保国有资产保值增值。制定出台《关于加快推进混合所有制经济发展的意见》，开展混合所有制试点工作，进一步规范行政许可，使更多的经营领域面向社会资本开放。

　　把创新投融资机制作为激发市场活力的有效手段。切实抓好深化投融资体制改革21条意见落实，有效破解企业融资难、融资贵的问题。规范税收优惠政策，加快消费税、资源税、房产税和营改增试点步伐。积极扶持投融资担保、小额贷款、投资基金等机构建设，推进交通银行、长安银行等市域内金融机构向县镇延伸，支持建立社区银行、村镇银行，力争年末全市贷款余额突破1000亿元。发挥财政投资控股有限公司作用，建立健全各类产业投资基金，推进城投集团转型发展和扩资增信。创新金融服务方式，积极探索开展PPP融资试点，解决基础设施和公共服务投入不足问题。支持科隆橡胶、康惠制药等企业上市，扩大直接融资规模。加快公共资源交易中心建设，鼓励引导非政府项目进入。强化金融市场监管和诚信体系建设，依法严厉打击非法集资行为，有效防范金融风险。

　　充分发挥科技创新在推动转型升级中的重要作用。认真落实加强科技创新工作《实施意见》，以建设国家创新型城市为抓手，深入推进"12351"科技创新工程，着力突破科研项目与产业发展脱节的瓶颈制约，促进企业与高等院校、科研院所协同创新，推动咸阳配套向咸阳制造、咸阳创造迈进。支持三原申报省级高新技术产业开发区，支持泾阳申报国家农业科技园区。实施质量强市和品牌兴市战略，加快培育中国驰名商标。

　　各位代表，发展是责任，改革是担当，我们将牢固树立创新意识、进取意识，围绕咸阳经济社会发展各项工作，真抓实干，砥砺前行，奋力书写咸阳民生改善、经济腾飞的新篇章！

　　四、加强政府自身建设

　　全面推进依法行政，持续改进工作作风，着力增强政府的公信力和执行力。

　　（一）强化法治意识，提升依法履职能力。严格按照宪法和法律规定的权限和程序办事，大力推动政府公务人员学法、懂法、守法、用法，把政府工作全面纳入法治轨道。加快推行权力清单、责任清单和负面清单制度，完善重大事项公众参与、专家论证、风险评估、合法性审查和集体讨论相结合的决策制度。切实规范行政执法行为，积极推进综合行政执法，着力解决权责交叉、多头执法问题。自觉接受人大及其常委会的法律监督、工作监督和政协的民主监督，主动接受公众监督和媒体监督。

　　（二）强化创新意识，提升服务发展能力。加强干部理论武装和专业知识培训，促进各级干部转变发展理念，成为经济转型发展的"推动者"和"操盘手"。注重借助智库和外脑，综合运用规划、产业政策等手段，推动产业结构调整和经济发展方式转变。坚持运用市场手段破解发展瓶颈制约，注重全要素聚集整合，提高做好经济工作的专业化、科学化水平。

　　（三）强化执行意识，提升推动落实能力。坚持勇于负重、实干克难，坚决克服守摊子思想和为官不为现象，满腔热情地为基层、为企业、为群众服务。坚持问题导向，围绕发展大事、改革要事、民生难事，以抓铁有痕、踏石留印的精神确保各项决策部署落实到位、执行到位。进一步完善对标管理体系，健全目标责任制管理、绩效考核、督查督办和责任追究制度，努力营造崇尚实干、恪尽职守、甘于奉献的工作氛围。

　　（四）强化自律意识，提升拒腐防变能力。巩固深化群众路线教育实践活动成果，推动作风建设常态化、长效化。持之以恒纠正"四风"，严格执行"八项规定"，压缩"三公经费"支出，推进公车改革。坚决落实党风廉政建设主体责任，坚持用制度管权管事管人，强化审计监督和行政监察，严肃查处腐败案件，形成干部不敢腐、不能腐、不想腐的有效机制。

　　各位代表，实干收获希望，拼搏铸就未来。让我们在市委的坚强领导下，励精图治，埋头苦干，为建设"四强三领先"西部强市而努力奋斗！
